# Factors Associated with Loss to Follow-up during Treatment for Multidrug-Resistant Tuberculosis, the Philippines, 2012–2014

**DOI:** 10.3201/eid2203.151788

**Published:** 2016-03

**Authors:** Thelma E. Tupasi, Anna Marie Celina G. Garfin, Ekaterina V. Kurbatova, Joan M. Mangan, Ruth Orillaza-Chi, Leilani C. Naval, Glenn I. Balane, Ramon Basilio, Alexander Golubkov, Evelyn S. Joson, Woo-jin Lew, Vivian Lofranco, Mariquita Mantala, Stuart Pancho, Jesus N. Sarol

**Affiliations:** Tropical Disease Foundation, Inc., Makati City, the Philippines (T.E. Tupasi, L.C. Naval, G.I. Balane, E.S. Joson, J.N. Sarol Jr.);; Department of Health, Manila, the Philippines (A.M.C.G. Garfin, R. Basilio);; Centers for Disease Control and Prevention, Atlanta, Georgia, USA (E.V. Kurbatova, J.M. Mangan);; Philippine Business for Social Progress–Innovations and Multisectoral Partnership to Achieve Control of Tuberculosis Project, Manila (R. Orillaza-Chi);; US Agency for International Development (USAID), Washington, DC, USA (A. Golubkov);; World Health Organization Philippines, Manila (W.-j. Lew);; The Lung Center of the Philippines, Manila (V. Lofranco, S. Pancho);; Technical Assistance to the Countries–USAID-funded activity, Manila (M. Mantala)

**Keywords:** multidrug-resistant tuberculosis, MDR TB, patient compliance, risk factors, tuberculosis and other mycobacteria, the Philippines, treatment, follow-up, TB, side effects

## Abstract

To identify factors associated with loss to follow-up during treatment for multidrug-resistant (MDR) tuberculosis (TB) in the Philippines, we conducted a case–control study of adult patients who began receiving treatment for rifampin-resistant TB during July 1–December 31, 2012. Among 91 case-patients (those lost to follow-up) and 182 control-patients (those who adhered to treatment), independent factors associated with loss to follow-up included patients’ higher self-rating of the severity of vomiting as an adverse drug reaction and alcohol abuse. Protective factors included receiving any type of assistance from the TB program, better TB knowledge, and higher levels of trust in and support from physicians and nurses. These results provide insights for designing interventions aimed at reducing patient loss to follow-up during treatment for MDR TB.

## Abstract 2

Most commonly reported was medication side effects or fear of side effects.

The Philippines is 1 of 22 countries considered to have a high burden of tuberculosis (TB) (*1*), including multidrug-resistant (MDR) TB (resistant to isoniazid and rifampin) (*1*). Compared with treatment for drug-susceptible TB, treatment for MDR TB is longer, more expensive, and less effective, and it causes more medication side effects (*2*–*5*). Resistance to anti-TB drugs has been detected in all regions of the Philippines; an estimated 8,500 MDR TB cases occurred in 2013 (*6*).

Programmatic Management of Drug-resistant Tuberculosis (PMDT) was jointly initiated in the Philippines in October 2000 by the private Makati Medical Center in Metro Manila and the Tropical Disease Foundation, Inc., in collaboration with the National TB Control Program and the local government unit, as the first directly observed therapy (DOTS)–plus pilot project for the management of MDR TB approved by the Green Light Committee (*7*). In 2003, a grant proposal from the Philippines for Round 2 of the Global Fund to Fight AIDS, Tuberculosis and Malaria included treatment for 500 patients with MDR TB (National Tuberculosis Control Program, the Philippines, 2013 Aug 25–Sep 6. Report of the Joint Program Review; 2013 Sep 30, unpub. data). This funding was approved and subsequently expanded to 2,500 MDR TB patients approved to receive treatment according to a Round 5 proposal. In 2010, a new coordination team for PMDT was established by the National TB Control Program/Department of Health; the Lung Center of the Philippines was the implementing arm for PMDT. As of September 2014, a total of 44 PMDT health facilities were located in 16 of 17 regions. The annual number of patients with drug-resistant TB who began receiving treatment under PMDT increased from 191 in the 2005 cohort to 2,056 in the 2012 cohort. Despite substantial progress made by PMDT in the Philippines, the proportion of patients for whom treatment was successful decreased from 73% in the 2005 cohort to 46% in the 2010 cohort, while the proportion of loss to follow-up increased from 13% to 38%, respectively (*8*). Even with recent efforts to improve retention of patients receiving treatment for TB (e.g., providing transportation allowance, financial incentives at treatment milestones, food baskets, and halfway houses for patients from remote areas), the proportion of patients lost to follow-up remains substantial.

An effective approach to reducing loss to follow-up during treatment for MDR TB is needed (National Tuberculosis Control Program, the Philippines, 2013 Aug 25–Sep 6. Report of the Joint Program Review; 2013 Sep 30, unpub. data), especially in light of data from a prospective multinational study demonstrating that among MDR TB patients lost to follow-up, almost a third had extensively drug-resistant or pre–extensively drug-resistant TB when treatment was started; drug resistance was acquired during treatment by an additional 12% (*9**,**10*). In addition, a third of those lost to follow-up remained culture-positive at last contact, enabling community transmission of strains with more extensive resistance (*10*). However, most studies of loss to follow-up were done retrospectively, through medical record reviews (*11*–*17*), and lacked a theoretical framework. Specific reasons why patients in the Philippines are lost to follow-up during MDR TB treatment are limited and based primarily on the views of healthcare providers. We aimed to determine which individual, diagnosis and treatment, interpersonal, healthcare setting, and social factors were significantly associated with patient loss to follow-up during MDR TB treatment in the Philippines.

## Methods

### Study Design and Patient Population

We conducted a case–control study among adult patients (>18 years of age) with confirmed MDR or rifampin-resistant TB for whom treatment was initiated during July 1–December 31, 2012, at PMDT treatment facilities in the Philippines. We excluded from study inmates, children <18 years of age, patients enrolled in pharmaceutical clinical trials, and patients who had a major psychiatric disorder or were physically incapacitated. 

The study was approved by the institutional review board of the Tropical Disease Foundation, Inc., the Lung Center of the Philippines–Ethics Review Committee, and the Ethics Research Committee of the Philippine Tuberculosis Society, Inc. The US Centers for Disease Control and Prevention (CDC) determined that CDC staff involvement did not constitute engagement in human subject research and that submission for CDC institutional review board review was not required.

In the Philippines, the standardized treatment regimen for MDR TB is pyrazinamide, kanamycin, levofloxacin, prothionamide, and cycloserine; the intensive phase lasts >6 months and the continuation phase an additional >12 months. For this study, case-patients were defined as patients who were lost to follow-up from MDR TB treatment (i.e., patients whose treatment was interrupted for >2 consecutive months) (*18*). Those who later returned (after being considered lost to follow-up) at the time of interview were eligible for inclusion in the study as case-patients. Control-patients were defined as patients who had continued treatment for MDR TB for >12 months or who had a documented treatment outcome of cured, completed, or failed (*18*). Data collection and interviews were conducted from April 14 through July 31, 2014; thus, all control-patients were receiving MDR TB treatment for >15 months.

**Figure 1:**
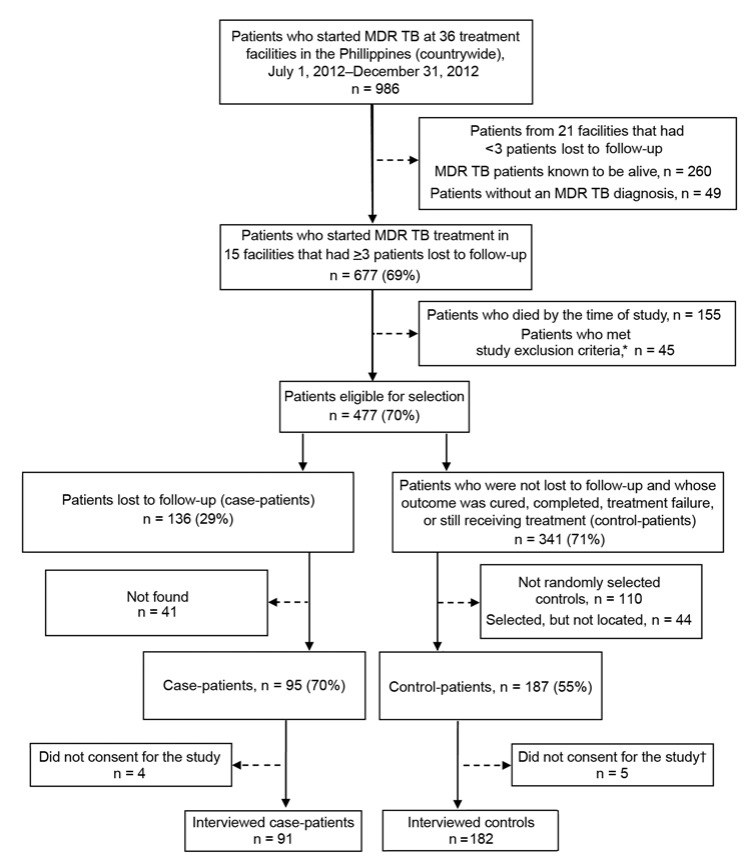
Selection of participants for study of loss to follow-up during treatment for multidrug resistant tuberculosis (MDR TB) in the Philippines, 2012–2014. *Study exclusion criteria were incarceration, age <18 years, enrollment in pharmaceutical clinical trials, and major psychiatric disorder or physical incapacitation. †Control-patients who did not give consent for the study were replaced by other randomly selected eligible patients.

Case-patients were identified by review of MDR TB registers. The number of patients who were lost to follow-up per treatment facility was assessed; centers with >3 patients with drug-resistant TB who had been lost to follow-up by January 1, 2014, and who were not known to have died, were selected for logistical reasons. Field study staff attempted to find all eligible patients who were lost to follow-up and invite them to participate in the study. Two control-patients were randomly selected for each enrolled case-patient from the same PMDT treatment facilities at which treatment was initiated for case-patients. Of 986 eligible patients, a total of 273 were enrolled: 91 case-patients and 182 control-patients (Figure 1).

**Figure 2:**
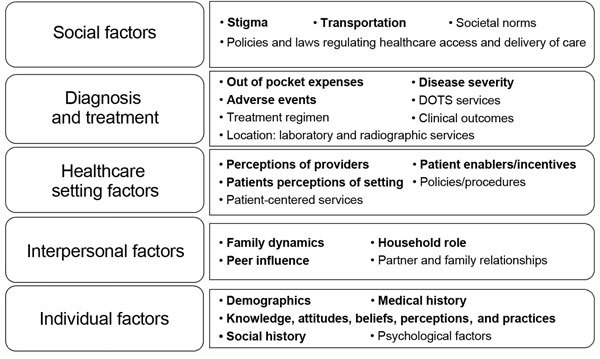
Social ecologic model used to identify factors influencing loss to follow-up during treatment for multidrug resistant tuberculosis in the Philippines, 2012–2014. Boldface indicates data collected through patient interviews and medical record abstractions. DOTS, directly observed therapy.

To characterize factors associated with loss to follow-up during MDR TB treatment, we followed a 5-level social ecologic model (*19**,**20*) that focuses on individual and environmental factors that affect health outcomes: 1) individual factors; 2) interpersonal factors; 3) healthcare setting factors, such as individual experiences with services and relationships within the setting; 4) diagnosis and treatment factors; and 5) social factors (Figure 2). To operationalize each category of factors and develop data collection forms, investigators reviewed TB literature and a 2013 Joint Program Review of the National Tuberculosis Control Program in the Philippines, which was led by the World Health Organization (WHO), and solicited input from experts within the country.

### Data Collection

Clinical and laboratory data were abstracted from participants’ medical records by using standard data collection forms. In-depth interviews with a series of closed- and open-ended questions were conducted to collect information about the following: demographics, social history, adverse drug reaction experiences, TB knowledge, perceived barriers to treatment completion, self-efficacy to adhere to treatment (*21*), values and expectancies associated with treatment; psychosocial factors (e.g., stigma, sources of emotional support, and family dynamics), financial support, perceptions of the healthcare setting, their diagnosis, their prescribed treatment, impressions of and feedback for the PMDT program regarding the current TB enabler package and patient-centered activities, and interventions under consideration. Cumulative scores were calculated from items focused on 1) patient perceptions of disease severity and TB knowledge; 2) expected outcomes, treatment self-efficacy; 3) patient-reported social support from family and friends; 4) trust in, rapport with, and support from health center staff; and 5) stigma (Table 1). Case-patients were asked to report their primary reason for stopping treatment. (Interview and scoring instruments are available from T.E.T.)

**Table 1:** Calculation of summary scores in study of loss to follow-up during treatment for multidrug-resistant TB, the Philippines, 2012–2014*

Score type	Score calculation	Score interpretation
General TB knowledge, including understanding of severity of the disease and susceptibility to the disease	Participants were asked 15 questions that focused on 1) the severity of the TB problem in their community, 2) TB transmission and morbidity/mortality, and 3) TB treatment. Each item answered correctly was awarded 1 point. Incorrect answers or “Not sure” responses received 0 points. The summary score was extrapolated onto a scale of 100 and reported as a percentage by using the following formula: General TB knowledge score = (total points earned/15) × 100 (i.e., score is calculated on a scale of 0–100%).	A higher score may indicate greater TB knowledge and greater perceived severity and susceptibility to the disease.
Expectations related to TB and its treatment	Participants were asked 5 interview questions aimed at determining their concerns for passing TB to loved ones, relapsing, and developing worsening drug resistance. Possible range of scores 5–15.	A higher score may indicate greater concerns or an expectation that TB could cause problems in the future. In addition to factors such as knowledge, attitudes, and beliefs, expected outcomes can determine a person’s actions. These expectations may be derived from 1) previous experiences, 2) observing or hearing about others in similar situations, 3) persuasive conversations, or 4) emotional or physical responses.
Self-efficacy (or confidence) to adhere to treatment at the time treatment was about to begin	Eight interview questions were included in the self-efficacy questionnaire. “Very confident” = 3 points, “A little confident” = 2 points, “unsure” = 1 point, and “I knew I could not do this” = 0 points. The score for each item would be added together to calculate a cumulative self-efficacy score. Possible range of scores 0–24 points.	A higher score may indicate a high degree of self-reported self-efficacy for adhering to treatment regimen, coping with the treatment, and meeting with DOT staff when about to start treatment.
Social support from family and friends	Score was based on responses to 3 interview questions with possible range of scores 3–15.	Lower scores may indicate less support.
Trust in, rapport with, and support from physicians and nursing staff	An overall score was based on 22 items grouped together. Possible range of scores 22–110.	A higher score may indicate a greater level of trust, rapport, and perceived support. Items were separated by topic, and separate scores were also calculated for participants’ 1) trust in, and rapport with physicians (13 questions); 2) trust in, and rapport with nurses (5 questions); and 3) perceived support from health center staff (4 questions).
Patient self-stigmatization	A cumulative score for stigma was based on 2 interview questions. Possible range of scores 1–10.	A higher score may indicate less stigma.

*DOTS, directly observed therapy; TB, tuberculosis.

### Data Analysis

Data were entered in a Microsoft (Redmond, WA, USA) Access electronic database. Statistical analyses were performed by using SAS software version 9.3 (SAS Institute Inc., Cary, NC, USA). Thematic qualitative analysis was conducted by using Microsoft Excel software.

We assessed associations between individual, interpersonal, healthcare setting, diagnosis and treatment, and social factor data and an outcome of lost to follow-up. For continuous variables, we compared means (SDs) or medians (percentiles) or both, depending on the underlying distributions. The proportions of patients with characteristics of interest were compared between case-patients and control-patients by χ^2^ or Fisher exact tests, as appropriate. We calculated odds ratios with corresponding 95% CIs. To identify independent factors associated with loss to follow-up, we performed multivariable logistic regression analyses. The initial multivariable model included covariates with epidemiologic, biological, or statistical associations with the dependent variable. We evaluated effect modification and confounding in the full model, and then we performed backward elimination to improve precision of the estimates (*22*). All tests were 2-sided, and a p value of <0.05 was considered statistically significant.

## Results

Mean ± SD age of the 273 study participants at start of MDR TB treatment was 39 ± 13 (median 40, range 16–68) years; 164 (60.1%) were male. An HIV test result was recorded for 56 (20.5%) of the 273 patients; 2 (3.5%) were HIV positive. All patients had pulmonary TB, and 35% had cavitary TB.

Most (70 [77.8%]) of the 90 case-patients for whom information on length of treatment was available were lost to follow-up during the intensive phase of treatment. Mean ± SD time receiving MDR TB treatment for case-patients was 7.8 ± 3.4 months (median 7 months; 25th percentile 4 months; 75th percentile 11 months) and for control-patients was 19.8 ± 1.7 months (median 20 months; 25th percentile 19 months; 75th percentile 21 months). Most (121 [66.5%]) of the 182 control-patients were still receiving treatment at the time of interview. Among 61 control-patients for whom treatment outcome was available, 35 (57.4%) experienced cure, 24 (39.3%) completed treatment, and 2 (3.3%) experienced treatment failure.

Univariate analysis indicated that individual factors significantly associated with loss to follow-up included older age (mean ± SD age 42 ± 13 years for case-patients vs. 38 ± 12 years for control-patients; p = 0.028); tobacco smoking (odds ratio [OR] 2.86, 95% CI 1.65–4.97); alcohol abuse (OR 1.93, 95% CI 1.09–3.40); and residence in an urban slum (OR 0.52, 95% CI 0.29–0.91) (Table 2). General knowledge of TB was significantly lower among case-patients than among control-patients; knowledge included understanding of the severity of and susceptibility to the disease (mean ± SD score 67.8 ± 16.3 vs. 74.3 ± 13.8, respectively; p<0.001), but recall for self-confidence for adhering to treatment at the time of treatment start was significantly higher among case-patients (4.9 ± 5.3 vs. 2.44 ± 3.8, respectively, p<0.001) (*20*). 

**Table 2:** Univariate analysis of Individual factors associated with loss to follow-up during treatment for multidrug-resistant TB, the Philippines, 2012–2014*

Factor	Total†	Case-patients‡	Control-patients‡	Odds ratio (95% CI)	p value
Data from review of medical records					
Demographics					
Sex					
M	164	60 (65.9)	104 (57.1)	1.45 (0.86–2.45)	0.16
F	109	31 (34.1)	78 (42.9)	1.00	
Age	273	41.6 (13.2)§	38.0 (12.5)§	1.02 (1.00–1.04)¶	**0.028**
Social history					
Tobacco smoking					
Current, past	153	65 (73)	88 (48.6)	2.86 (1.65–4.97)	**<0.001**
Never	117	24 (27)	93 (51.4)	1.00	
Alcohol abuse					
Current, past	175	66 (75)	109 (60.9)	1.93 (1.09–3.4)	**0.02**
Never	92	22 (25)	70 (39.1)	1.00	
Drug abuse					
Current, past	52	19 (22.4)	33 (18.6)	1.26 (0.67–2.37)	0.48
Never	210	66 (77.6)	144 (81.4)	1.00	
Clinical information					
No. previous TB episodes	273	1.71 (0.90)§	1.62 (1.07)§	1.10 (0.86–1.40)¶	0.449
BMI					
<18.5	146	51 (56)	95 (52.2)	1.17 (0.7–1.94)	0.55
>18.5	127	40 (44)	87 (47.8)	1.00	
Cavitary TB disease					
Yes	97	31 (44.3)	66 (41)	1.14 (0.65–2.02)	0.64
No	134	39 (55.7)	95 (59)	1.00	
Smear-positive at treatment start					
Yes	219	70 (82.4)	149 (85.1)	0.81 (0.41–1.63)	0.56
No	41	15 (17.6)	26 (14.9)	1.00	
Data from patient interviews					
Total no. persons residing in household	272	5.12 (2.79)§	5.53 (2.95)§	1.94 (0.63–5.99)¶	0.27
Residence Comparison 1					
Rural area	40	15 (23.4)	25 (24.5)	0.94 (0.45–1.96)	0.88
Urban slum	126	49 (76.6)	77 (75.5)	1.00	
Residence Comparison 2					
Urban area	105	26 (34.7)	79 (50.6)	0.52 (0.29–0.91)	**0.02**
Urban slum	126	49 (65.3)	77 (49.4)	1.00	
Paid employment before starting treatment					
Yes	126	49 (54.4)	77 (42.3)	1.63 (0.98–2.71)	0.06
No	146	41 (45.6)	105 (57.7)	1.00	
Employed before starting treatment but had to quit#					
Yes	90	35 (79.5)	55 (83.3)	0.78 (0.29–2.07)	0.61
No	20	9 (20.5)	11 (16.7)	1.00	
Employed before starting treatment but fired/asked to take leave of absence#					
Yes	10	5 (35.7)	5 (31.3)	1.22 (0.27–5.59)	0.80**
No	20	9 (64.3)	11 (68.8)	1.00	
Family sold belongings or household items (assets) to help pay expenses during TB treatment					
Yes	93	28 (31.8)	65 (35.7)	0.84 (0.49–1.44)	0.53
No	177	60 (68.2)	117 (64.3)	1.00	
Family borrowed money to cover costs due to TB illness					
Yes	181	60 (74.1)	121 (70.8)	1.18 (0.65–2.14)	0.58
No	71	21 (25.9)	50 (29.2)	1.00	
General TB knowledge††	272	67.81 (16.31)§	74.25 (13.78)§	0.97 (0.95–0.99)¶	**<0.001**
Expectations related to TB and TB treatment	272	11.01 (1.87)§	10.76 (1.55)§	1.10 (0.94–1.28)¶	0.28
Self-efficacy (or confidence) to adhere to treatment at the time treatment was about to begin	272	4.91 (5.28)§	2.44 (3.77)§	1.13 (1.06–1.19)¶	**<0.001**

*Boldface indicates significance. BMI, body mass index; TB, tuberculosis.  †Total reflects number of patients for whom data or responses for each respective category were available.  ‡No. (%) unless noted otherwise.  §Mean (SD).  ¶Odds ratio is per 1 unit increase. #Of 126 patients who had paid employment before starting treatment, 90 reported that they subsequently “had to quit” and 10 reported that they had subsequently been “fired/asked to take leave of absence.” **Fisher exact test. ††Such as understanding of disease severity, susceptibility, scale 0%–100%).

Univariate analysis also indicated that the only interpersonal factor significantly associated with loss to follow-up was social support from family and friends. Scores were lower among case-patients than among control-patients (mean ± SD score 12.1 ± 3.4 vs. 12.9 ± 3.0, respectively, p = 0.047) (Table 3).

**Table 3:** Univariate analysis of interpersonal factors associated with loss to follow-up during treatment for multidrug-resistant TB, the Philippines, 2012–2014*

Characteristic, from data from patient interviews	Total	Case-patients†	Control-patients†	Odds ratio (95% CI)	p value
Head of household‡					
Yes	91	37 (41.1)	54 (29.7)	1.65 (0.98–2.8)	0.06
No	181	53 (58.9)	128 (70.3)	1.00	
In charge of household budget					
Yes	96	32 (35.6)	64 (35.2)	1.02 (0.6–1.72)	0.95
No	176	58 (64.4)	118 (64.8)	1.00	
Social support from family and friends	271	12.07 (3.35)§	12.87 (2.99)§	0.92 (0.85–1.00)¶	**0.047**

*Boldface indicates significance. TB, tuberculosis. †No (%) unless noted otherwise.  ‡Provided more than half the cost of keeping up a home the year before becoming sick with TB.  §Mean (SD). ¶Odds ratio is per 1 unit increase.

Among healthcare setting factors, case-patients reported having received any type of assistance from the TB program significantly less often than control-patients: overall (OR 0.11, 95% CI 0.04–0.29), food (OR 0.46, 95% CI 0.27–0.79), free medications for treatment of adverse drug reactions (OR 0.28, 95% CI 0.16–0.48), or money for transportation (OR 0.19, 95% CI 0.1–0.37) **(**Table 4). Scores for trust in, rapport with, and support from physicians and nursing staff were significantly lower among case-patients than among control-patients (mean ± SD score 81.9 ± 15.6 vs. 90.7 ± 7.8, respectively; p<0.001), as were scores for trust in and rapport with physicians (56.0 ± 11.5 vs. 62.2 ± 5.4, respectively; p<0.001), trust in and rapport with nurses (21.8 ± 3.7 vs. 23.8 ± 2.4, respectively; p<0.001), and information and support received from healthcare center staff (7.5 ± 1.7 vs. 8.4 ± 1.2, respectively; p<0.001).

**Table 4:** Univariate analysis of healthcare setting factors associated with loss to follow-up during treatment for multidrug-resistant TB, the Philippines, 2012–2014*

Characteristic, from data from patient interviews	Total	Case-patients†	Control-patients†	Odds ratio (95% CI)	p value
Treatment center provided financial assistance or other items to facilitate treatment adherence					
Yes	245	69 (76.7)	176 (96.7)	0.11 (0.04–0.29)	**<0.001**
No	27	21 (23.3)	6 (3.3)	1.00	
Types of assistance provided					
Food products					
Yes	114	27 (29.7)	87 (47.8)	0.46 (0.27–0.79)	**0.004**
No	159	64 (70.3)	95 (52.2)	1.00	
Housing assistance					
Yes	15	6 (6.6)	9 (4.9)	1.36 (0.47–3.94)	0.57
No	258	85 (93.4)	173 (95.1)	1.00	
Free medications to treat side effects from anti-TB drugs					
Yes	196	49 (53.8)	147 (80.8)	0.28 (0.16–0.48)	**<0.001**
No	77	42 (46.2)	35 (19.2)	1.00	
Money for transportation					
Yes	224	59 (64.8)	165 (90.7)	0.19 (0.1–0.37)	**<0.001**
No	49	32 (35.2)	17 (9.3)	1.00	
Trust in, rapport with, and support from physicians and nursing staff	273	81.88 (15.56)‡	90.73 (7.79)‡	0.93 (0.91–0.96)§	**<0.001**
Information and support from healthcare center staff	272	7.53 (1.68)‡	8.43 (1.22)‡	0.64 (0.52–0.78)§	**<0.001**

*Boldface indicates significance. TB, tuberculosis. †No (%) unless noted otherwise.  ‡Mean (SD).  §Odds ratio is per 1 point increase in cumulative score.

Among diagnosis and treatment factors, frequency of certain adverse drug reactions reported by patients did not differ significantly, except for less frequently reported diarrhea among case-patients (OR 0.49, 95% CI 0.29–0.85). However, scores for self-reported severity of adverse drug reactions were significantly higher among case-patients; reactions included vomiting (mean ± SD 5.23 ± 3.72 vs. 4.02 ± 3.46; p = 0.008), dizziness (5.48 ± 3.51 vs. 4.55 ± 3.14; p = 0.029), and fatigue or extreme tiredness (5.18 ± 3.56 vs. 4.14 ± 3.25; p = 0.017) (Tables 5, 6). Reported cost of travel to the treatment center during the intensive phase of treatment was significantly higher among case-patients than among control-patients (mean ± SD 98 ± 104 pesos vs. 71 ± 57 pesos, respectively, p = 0.035).

**Table 5:** Univariate analysis of diagnosis and treatment factors associated with loss to follow-up during treatment for multidrug-resistant TB, the Philippines, 2012–2014*

Characteristic, data from patient interviews	Total	Case-patients†	Control-patients†	Odds ratio (95% CI)	p value
Side effects during MDR TB treatment‡					
Nausea					
Yes	230	74 (81.3)	156 (85.7)	0.73 (0.37–1.42)	0.35
No	43	17 (18.7)	26 (14.3)	1.00	
Vomiting					
Yes	210	74 (81.3)	136 (74.7)	1.47 (0.79–2.75)	0.22
No	63	17 (18.7)	46 (25.3)	1.00	
Diarrhea					
Yes	195	56 (61.5)	139 (76.4)	0.49 (0.29–0.85)	**0.01**
No	78	35 (38.5)	43 (23.6)	1.00	
Headache					
Yes	212	68 (75.6)	144 (79.1)	0.82 (0.45–1.48)	0.50
No	60	22 (24.4)	38 (20.9)	1.00	
Sleep disturbances					
Yes	231	75 (83.3)	156 (85.7)	0.83 (0.42–1.67)	0.61
No	41	15 (16.7)	26 (14.3)	1.00	
Tingling/pain in hands or feet					
Yes	187	55 (61.1)	132 (72.5)	0.6 (0.35–1.02)	0.06
No	85	35 (38.9)	50 (27.5)	1.00	
Hearing problems					
Yes	202	68 (75.6)	134 (73.6)	1.11 (0.62–1.98)	0.73
No	70	22 (24.4)	48 (26.4)	1.00	
Dizziness					
Yes	231	75 (83.3)	156 (85.7)	0.83 (0.42–1.67)	0.61
No	41	15 (16.7)	26 (14.3)	1.00	
Nervousness/anxiety					
Yes	160	51 (56.7)	109 (59.9)	0.88 (0.53–1.46)	0.61
No	112	39 (43.3)	73 (40.1)	1.00	
Pain in joints					
Yes	221	68 (75.6)	153 (84.1)	0.59 (0.31–1.09)	0.09
No	51	22 (24.4)	29 (15.9)	1.00	
Vision problems					
Yes	155	53 (58.9)	102 (56)	1.12 (0.67–1.87)	0.66
No	117	37 (41.1)	80 (44)	1.00	
Fatigue/extreme tiredness					
Yes	208	70 (77.8)	138 (75.8)	1.12 (0.61–2.04)	0.72
No	64	20 (22.2)	44 (24.2)	1.00	
Participant travel expenses					
Cost to travel to treatment center, intensive phase of treatment	239	97.58 (103.64)§	70.83 (57.24)§	1.00 (1.00–1.01¶	**0.035**
Cost to travel to treatment center, continuation phase of treatment	138	67.93 (65.24)§	52.46 (39.37)§	1.01 (0.00–1.02)¶	0.383
Source of funds to travel to/from treatment center, intensive phase of treatment					
Own/personal money					
Yes	203	68 (74.7)	135 (74.2)	1.03 (0.58–1.83)	0.92
No	70	23 (25.3)	47 (25.8)	1.00	
TB program funds					
Yes	183	48 (52.7)	135 (74.2)	0.39 (0.23–0.66)	**<0.001**
No	90	43 (47.3)	47 (25.8)	1.00	
Borrowed money					
Yes	99	38 (41.8)	61 (33.5)	1.42 (0.85–2.39)	0.18
No	174	53 (58.2)	121 (66.5)	1.00	

*Boldface indicates significance. TB, tuberculosis. †No. (%) unless noted otherwise. ‡Participants were asked to rate severity of medication side effects on a scale of 1–10 (0 = absence of symptoms; 1 = very mild, 10 = extremely severe). §Mean (SD).  ¶Odds ratio is per 1 point increase in cost unit.

**Table 6:** Rating of the severity of medication side effects experienced during treatment for multidrug-resistant TB, the Philippines, 2012–2014*

Side effect	Total	Case-patients, mean (SD) score	Control-patients, mean (SD) score	Odds ratio (95% CI)†	p value
Nausea	273	5.05 (3.51)	4.42 (3.18)	1.06 (0.98–1.14)	0.136
Vomiting	273	5.23 (3.72)	4.02 (3.46)	1.10 (1.02–1.18)	**0.008**
Diarrhea	273	3.34 (3.41)	3.74 (3.18)	0.96 (0.89–1.04)	0.345
Headache	272	4.37 (3.36)	4.20 (3.23)	1.02 (0.94–1.10)	0.689
Sleep disturbances	272	5.23 (3.59)	5.31 (3.26)	0.99 (0.92–1.07)	0.854
Tingling/pain in hands or feet	272	3.58 (3.53)	4.14 (3.56)	0.96 (0.89–1.03)	0.218
Hearing problems	272	4.49 (3.47)	4.13 (3.46)	1.03 (0.96–1.11)	0.417
Dizziness	272	5.48 (3.51)	4.55 (3.14)	1.09 (1.01–1.18)	**0.029**
Nervousness/anxiety	272	3.26 (3.34)	3.01 (3.16)	1.02 (0.95–1.11)	0.548
Skin problems or rash	272	2.70 (3.26)	2.69 (3.22)	1.00 (0.93–1.08)	0.975
Joint pain	272	5.14 (3.57)	5.49 (3.34)	0.97 (0.90–1.04)	0.427
Vision problems	272	3.10 (3.07)	2.80 (3.05)	1.03 (0.95–1.12)	0.45
Fatigue/extreme tiredness	272	5.18 (3.56)	4.14 (3.25)	1.10 (1.02–1.18)	**0.017**

*Boldface indicates significance. TB, tuberculosis. †Odds ratio is per 1 point increase in severity rating score.

Univariate analysis indicated that social factors significantly associated with loss to follow-up were self-reported lack of time to go to the treatment facility (OR 2.23, 95% CI 1.1–4.54) and absence of someone to accompany the patient to the treatment facility during the intensive phase of treatment (OR 1.97, 95% CI 1.06–3.65). Scores reflecting patient self-stigmatization among case-patients and control-patients did not differ significantly (p = 0.10) (Table 7).

**Table 7:** Univariate analysis of social factors associated with loss to follow-up during treatment for multidrug-resistant TB, the Philippines, 2012–2014*

Category	Total	Case-patients†	Control-patients†	Odds ratio (95% CI)	p value
Distance between participant's home and treatment center, intensive phase of treatment					
Comparison 1					
0 to <1 km	23	7 (23.3)	16 (31.4)	0.67 (0.24–1.87)	0.44
1 to <5 km (referent)	58	23 (76.7)	35 (68.6)	1.00	
Comparison 2					
5 to <10 km	46	13 (36.1)	33 (48.5)	0.6 (0.26–1.37)	0.23
1 to <5 km (referent)	58	23 (63.9)	35 (51.5)	1.00	
Comparison 3					
>10 km	104	38 (62.3)	66 (65.3)	0.88 (0.45–1.7)	0.69
1 to <5 km (referent)	58	23 (37.7)	35 (34.7)	1.00	
Comparison 4					
Not sure/don't know	42	10 (30.3)	32 (47.8)	0.48 (0.2–1.15)	0.10
1 to <5 km (referent)	58	23 (69.7)	35 (52.2)	1.00	
Usual mode of transportation/transportation used to cover the greatest distance traveling to the treatment center, intensive phase of treatment					
Walk					
Yes	37	19 (20.9)	18 (9.9)	2.4 (1.19–4.85)	**0.01**
No	236	72 (79.1)	164 (90.1)	1.00	
Public transportation					
Yes	239	79 (86.8)	160 (87.9)	0.91 (0.43–1.92)	0.80
No	34	12 (13.2)	22 (12.1)	1.00	
Personal vehicle					
Yes	16	6 (6.6)	10 (5.5)	1.21 (0.43–3.45)	0.72
No	257	85 (93.4)	172 (94.5)	1.00	
Major challenges when traveling to the treatment center, intensive phase of treatment					
The center was far away					
Yes	141	52 (57.8)	89 (48.9)	1.43 (0.86–2.38)	0.17
No	131	38 (42.2)	93 (51.1)	1.00	
Did not always have money for transportation					
Yes	193	71 (78)	122 (67.4)	1.72 (0.96–3.08)	0.07
No	79	20 (22)	59 (32.6)	1.00	
Did not have the time to go for treatment					
Yes	36	18 (19.8)	18 (9.9)	2.23 (1.1–4.54)	**0.02**
No	236	73 (80.2)	163 (90.1)	1.00	
Going for treatment caused problems with work or school					
Yes	81	33 (36.3)	48 (26.5)	1.58 (0.92–2.71)	0.10
No	191	58 (63.7)	133 (73.5)	1.00	
Did not have anyone to go with					
Yes	52	24 (26.4)	28 (15.4)	1.97 (1.06–3.65)	**0.03**
No	221	67 (73.6)	154 (84.6)	1.00	
The center’s hours were not convenient					
Yes	23	11 (12.1)	12 (6.6)	1.95 (0.82–4.6)	0.12
No	250	80 (87.9)	170 (93.4)	1.00	
Minutes to travel from home to treatment center, intensive phase of treatment	272	51.00 (43.56)‡	54.16 (45.03)‡	1.00 (0.99–1.00)§	0.583
Minutes to travel from home to treatment center, continuation phase of treatment	198	22.25 (15.93)‡	31.07 (36.94)‡	0.99 (0.97–1.01)§	0.057
Patient self-stigmatization	272	6.20 (2.76)‡	5.66 (2.44)‡	1.09 (0.98–1.20)§	0.104

*Boldface indicates significance. TB, tuberculosis. †No. (%) unless noted otherwise. ‡Mean (SD).  §Odds ratio is per 1 point increase in unit.

Independent factors positively associated with loss to follow-up were alcohol abuse (OR 2.84, 95% CI 1.39–5.80) and patient higher self-rating of vomiting severity (OR 1.10, 95% CI 1.01–1.21, per 1 point in severity rating score). Factors protective against loss to follow-up were receipt of any type of assistance from the TB program (OR 0.09, 95% CI 0.03–0.25); better general TB knowledge (OR 0.97, 95% CI 0.95–0.99, per 1 point in cumulative score); and higher levels of trust in, rapport with, and support from physicians and nursing staff (OR 0.93, 95% CI 0.90–0.96, per 1 point in cumulative score) (Table 8).

**Table 8:** Multivariable analysis of factors associated with loss to follow-up during treatment for multidrug-resistant TB, the Philippines, 2012–2014*

Social ecologic model level, factor	Odds ratio (95% CI)	p value
Personal factors		
Score TB knowledge	0.97 (0.95–0.99)†	0.003
Alcohol abuse	2.84 (1.39–5.80)	0.004
Healthcare setting factors		
Received assistance from TB program	0.09 (0.03–0.25)	<0.001
Score trust/rapport with healthcare worker	0.93 (0.90–0.96)†	<0.001
Diagnosis and treatment factors		
Self-rated severity of vomiting as adverse drug reaction	1.10 (1.01–1.21)‡	0.03

*TB, tuberculosis.  †Odds ratio is per 1 point increase in cumulative score. ‡Odds ratio is per 1 point increase in severity rating score.

The primary reason for stopping treatment, most commonly reported by case-patients, was medication side effects or the fear of side effects, reported by 52 (58%) of 89 case-patients who responded to this question. The 2 other most commonly self-reported reasons for loss to follow-up were need to work and financial problems, reported by 25 (28%) of 89 patients, and lack of money for transportation to the treatment facility, reported by 18 (20%) of 89 patients. 

## Discussion

This large study, guided by a 5-level theoretical social ecologic model, demonstrated that loss to follow-up from MDR TB treatment in the Philippines was independently associated with 2 individual factors (general TB knowledge and alcohol abuse), 2 healthcare setting factors (receiving any type of assistance from the TB program and levels of trust in, rapport with, and support from physicians, nursing staff and other healthcare workers in the treatment facilities), and 1 diagnosis and treatment factor (higher self-rated severity of vomiting as an adverse drug reaction). Multivariable analysis did not identify any interpersonal or social factors associated with loss to follow-up. The most commonly reported primary reason for loss to follow-up was medication side effects or fear of side effects.

This study demonstrated that general TB knowledge was significantly lower among case-patients than among control-patients. Although nonadherence to treatment rarely results from patient apathy, patients’ lack of knowledge of their medical condition and its treatment is associated with poor health outcomes (*23*). For this reason, patient education is a valuable component of TB control. A recent systematic review and meta-analysis demonstrated that provision of patient education was a strategy associated with lower rates of loss to follow-up (*24*). Among TB patients, interventions to improve general TB knowledge are significantly associated with better outcomes (*25*). Knowledge of the bacterial causation of TB, mode of transmission, diagnostic testing, meaning of test results, rationale for prolonged treatment, and effect of treatment interruptions should be clearly explained to patients and their loved ones. It is also crucial to educate patients about expected adverse events before starting treatment. Patient education that addresses commonly held misperceptions about TB may also discourage patients from consulting traditional healers, thereby avoiding delayed diagnosis and treatment (*26*).

Alcohol abuse was recorded in medical records at a significantly higher frequency for case-patients than for control-patients. The association between alcohol abuse or alcohol use disorders and loss to follow-up during MDR TB treatment has been demonstrated in multiple studies (*13*–*15*,*27*); 1 small randomized clinical trial demonstrated improved TB outcomes for patients in groups randomly assigned to receive naltrexone or behavioral counseling integrated into TB care (*28*). This finding, when combined with similar findings in other studies, calls for additional studies to assess the effect of using standard assessment tools to screen for alcohol dependence and of managing alcohol use as part of TB clinical services. 

Receiving assistance from the TB program, including such measures as covering the cost of transportation, food, and housing, was associated with improved treatment adherence. However, if the process of applying for financial assistance is long and difficult and if this assistance is not given in a timely manner or regularly, patients may abandon their efforts to adhere to treatment. Decentralization of treatment (i.e., the transfer of care from a centralized MDR TB treatment center to a community DOTS facility) was protective; odds of being lost to follow-up decreased by 10 times (*12*). This finding suggests that the decentralization of care into multiple treatment facilities closer to patients’ homes, which was used as part of the PMDT scale-up scheme in the Philippines, is a valid strategy for improving TB treatment patient retention. In addition to decentralization, the National TB Control Program began piloting the Community-Based PMDT Care Initiative in 2014 to provide a more accessible venue for management of MDR TB in the patient’s home. The effectiveness of this initiative in improving treatment adherence should be rapidly evaluated, and the evaluation results should be used for future program planning. All modalities for addressing patient barriers should be considered. Adherence to MDR TB treatment might be improved by providing sufficient and timely financial assistance to patients (especially for transportation) by augmenting current enablers and providing livelihood programs during and after MDR TB treatment through strategic multisectoral partnership.

We found that levels of trust in, rapport with, and support from physicians, nursing staff, and other caregivers in the treatment facilities were significantly lower among patients lost to follow-up than among control-patients. A higher degree of trust, good rapport, and support from providers has been shown to be associated with patients’ adherence to medical recommendations and with improvements to self-reported and objective measures of health (*29**–**31*). In a study by Holtz et al. in South Africa, the strongest individual risk factor for nonadherence to MDR TB treatment was having an unsatisfactory opinion about the attitude of the healthcare workers (*32*). Interventions focused on enhancing provider–patient mutual trust and respect are needed. Provider training with regard to active listening, health literacy, message-framing, motivational interviewing, communication skills for trust-building and sensitivity should be considered.

We found that patients’ higher rating of the severity of their vomiting was independently associated with loss to follow-up. Moreover, the most commonly reported reason for stopping treatment (58%) was medication side effects. However, frequency of adverse drug reactions reported by patients in interviews did not differ significantly between case-patients and control-patients (except that diarrhea symptoms were reported significantly less often by case-patients than by control-patients). Still, case-patients reported significantly higher subjectively perceived severity of vomiting, dizziness, and fatigue as medication side effects. These symptoms affect quality of life and interfere with the capacity to work and the ability to engage in activities of daily living. A study of 583 MDR TB patients in the Philippines who had undergone treatment during 1999–2006 showed that taking >5 drugs was significantly associated with loss to follow-up compared with taking 2–3 drugs (*12*). The authors interpreted the association between a higher drug burden and loss to follow-up as being related to more extensive drug resistance and competing risk for death among those patients. However, it also might be related to experiencing more side effects by the patients who received a higher number of toxic second-line drugs, which more likely led to stopping treatment, than by patients who received a lower number of drugs. Our study suggests that strict monitoring for, and appropriate treatment of, adverse drug reactions may help improve treatment adherence. Ancillary drugs must be included in procurement plans and made widely available at treatment facilities. Initial and refresher trainings for healthcare providers about management of adverse drug reactions and patient education about expected adverse drug reactions before treatment initiation may also help improve treatment adherence. Patients should understand the need for strict monitoring for adverse drug reactions and the availability of effective and free treatment for those reactions, especially reactions that are subjectively difficult for patients (e.g., nausea, vomiting, dizziness, and extreme fatigue).

Our study is subject to several limitations. As with any case–control study, it provides relatively weak empirical evidence. Because patient interviews were part of the protocol, deceased patients were excluded, which might have introduced survivor biases into our results. Almost a third of selected control-patients could not be located, which could bias the results in favor of selecting control-patients with a stronger relationship to the clinics. The retrospective nature of interviews is also subject to recall and exposure misclassification biases. Interview responses may reflect socially desirable answers rather than true thoughts and experiences. Some patients who were adherent to treatment at the time of the interview may be lost to follow-up at a later time (in this study, 66% of control-patients were still receiving treatment at the time of the interview); thus, outcome misclassification is possible. Identification of alcohol abuse was based on the records in medical charts without standardized assessment for each patient (such as the Alcohol Use Disorder Identification Test) (*33*). Previous studies have demonstrated that loss to follow-up from treatment was less in smaller cohorts (*24*) and was more with program scale-up (*34*). Thus, increased loss to follow-up may be related to healthcare setting structural factors such as insufficient number of facilities and providers or limited experience with management of MDR TB, but our study did not address those factors. 

Despite these limitations, our study provides useful data. The interviews captured patients’ perspectives and provided nuances that retrospective cohort studies lack (*11*,*12*,*15*–*17*). These data, along with medical record data, afford program leaders greater insight for improving services and designing patient-centered interventions to reduce loss to follow-up during MDR TB treatment in the Philippines. A revision of the PMDT strategy should address identified barriers to completing MDR TB treatment and implement actions that support patients’ adherence to treatment.

Technical Appendix Multivariable analysis used to assess factors associated with loss to follow-up during treatment for multidrug-resistant tuberculosis, the Philippines, 2012–2014.

## References

[1] World Health Organization. Countdown to 2015: global tuberculosis report 2013 supplement [cited 2015 Jul 24]. http://apps.who.int/iris/bitstream/10665/91542/1/WHO_HTM_TB_2013.13_eng.pdf

[2] Ahuja SD, Ashkin D, Avendano M, Banerjee R, Bauer M, Bayona JN, Multidrug resistant pulmonary tuberculosis treatment regimens and patient outcomes: an individual patient data meta-analysis of 9,153 patients. PLoS Med. 2012;9:e1001300. 10.1371/journal.pmed.1001300 22952439 PMC3429397

[3] Fitzpatrick C, Floyd K. A systematic review of the cost and cost effectiveness of treatment for multidrug-resistant tuberculosis. Pharmacoeconomics. 2012;30:63–80. 10.2165/11595340-000000000-00000 22070215

[4] Orenstein EW, Basu S, Shah NS, Andrews JR, Friedland GH, Moll AP, Treatment outcomes among patients with multidrug-resistant tuberculosis: systematic review and meta-analysis. Lancet Infect Dis. 2009;9:153–61. 10.1016/S1473-3099(09)70041-6 19246019

[5] Ramachandran G, Swaminathan S. Safety and tolerability profile of second-line anti-tuberculosis medications. Drug Saf. 2015;38:253–69. 10.1007/s40264-015-0267-y 25676682

[6] World Health Organization. Tuberculosis country profiles [cited 2015 Jul 24]. https://extranet.who.int/sree/Reports?op=Replet&name=%2FWHO_HQ_Reports%2FG2%2FPROD%2FEXT%2FTBCountryProfile&ISO2=PH&LAN=EN&outtype=pdf

[7] Tupasi TE, Quelapio MID, Orillaza RB, Alcantara C, Mira NRC, Abeleda MR, DOTS-Plus for multidrug-resistant tuberculosis in the Philippines: global assistance urgently needed. Tuberculosis (Edinb). 2003;83:52–8. 10.1016/S1472-9792(02)00072-0 12758189

[8] The Philippines Department of Health. Report of accomplishments and targets of the Programmatic Management for Drug-resistant Tuberculosis program in the Philippines; 2014 [cited 2015 Jul 24]. http://www.doh.gov/ph/

[9] Cegielski JP, Kurbatova E, van der Walt M, Brand J, Ershova J, Tupasi T, Multidrug-resistant tuberculosis treatment outcomes in relation to treatment, initial and acquired second-line drug resistance. Clin Infect Dis. 2015. Oct 27. pii: civ910. Epub ahead of print [cited 2015 Jul 24]. http://www.ncbi.nlm.nih.gov/pubmed/26508515 10.1093/cid/civ910 PMC4725381 26508515

[10] Kurbatova E, Caoili JC, Contreras C, Ershova J, Dalton T, Kvasnovsky C, Loss to follow up from multidrug-resistant tuberculosis treatment and acquired drug resistance. Presented at: 45th Union World Conference on Lung Health 2014; 2014 Oct 28–Nov 1; Barcelona, Spain.

[11] Gelmanova IY, Keshavjee S, Golubchikova VT, Berezina VI, Strelis AK, Yanova GV, Barriers to successful tuberculosis treatment in Tomsk, Russian Federation: non-adherence, default and the acquisition of multidrug resistance. Bull World Health Organ. 2007;85:703–11. 10.2471/BLT.06.038331 18026627 PMC2636414

[12] Gler MT, Podewils LJ, Munez N, Galipot M, Quelapio MID, Tupasi TE. Impact of patient and program factors on default during treatment of multidrug-resistant tuberculosis. Int J Tuberc Lung Dis. 2012;16:955–60. 10.5588/ijtld.11.0502 22584124 PMC4616015

[13] Jakubowiak WM, Bogorodskaya EM, Borisov SE, Danilova ID, Kourbatova EV. Risk factors associated with default among new pulmonary TB patients and social support in six Russian regions. Int J Tuberc Lung Dis. 2007;11:46–53 . 17217129

[14] Kendall EA, Theron D, Franke MF, van Helden P, Victor TC, Murray MB, Alcohol, hospital discharge, and socioeconomic risk factors for default from multidrug resistant tuberculosis treatment in rural South Africa: a retrospective cohort study. PLoS ONE. 2013;8:e83480. 10.1371/journal.pone.0083480 24349518 PMC3862731

[15] Kliiman K, Altraja A. Predictors and mortality associated with treatment default in pulmonary tuberculosis. Int J Tuberc Lung Dis. 2010;14:454–63 . 20202304

[16] Kuchukhidze G, Kumar AMV, de Colombani P, Khogali M, Nanava U, Blumberg HM, Risk factors associated with loss to follow-up among multidrug-resistant tuberculosis patients in Georgia. Public Health Action. 2014;4(Suppl 2):S41–6. 10.5588/pha.14.0048 PMC4547510 26393097

[17] Shringarpure KS, Isaakidis P, Sagili K, Baxi RK. Loss-to-follow-up on multidrug resistant tuberculosis treatment in Gujarat, India: the when and who of it. PLoS ONE. 2015;10:e0132543. 10.1371/journal.pone.0132543 26167891 PMC4500497

[18] World Health Organization (WHO). Definitions and reporting framework for tuberculosis–2013 revision [cited 2015 Jul 24]. http://apps.who.int/iris/bitstream/10665/79199/1/9789241505345_eng.pdf

[19] Centers for Disease Control and Prevention. Colorectal Cancer Control Program. Social ecological model of health promotion [cited 2014 Jan 8]. http://www.cdc.gov/cancer/crccp/sem.htm

[20] Golden SD, Earp JAL. Social ecological approaches to individuals and their contexts: twenty years of health education & behavior health promotion interventions. Health Educ Behav. 2012;39:364–72. 10.1177/1090198111418634 22267868

[21] Bandura A. Self-efficacy: toward a unifying theory of behavioral change. Psychol Rev. 1977;84:191–215. 10.1037/0033-295X.84.2.191 847061

[22] Kleinbaum DG, Klein M, Pryor ER. Logistic regression: a self-learning text. New York: Springer-Verlag; 2002. p. 161–226.

[23] US Department of Health and Human Services. Quick guide to health literacy [cited 2015 Nov 20]. http://health.gov/communication/literacy/quickguide/Quickguide.pdf

[24] Toczek A, Cox H, du Cros P, Cooke G, Ford N. Strategies for reducing treatment default in drug-resistant tuberculosis: systematic review and meta-analysis. Int J Tuberc Lung Dis. 2013;17:299–307. 10.5588/ijtld.12.0537 23211716

[25] M’imunya JM, Kredo T, Volmink J. Patient education and counselling for promoting adherence to treatment for tuberculosis. Cochrane Database Syst Rev. 2012;5:CD006591 . 22592714 10.1002/14651858.CD006591.pub2 PMC6532681

[26] Viney KA, Johnson P, Tagaro M, Fanai S, Linh NN, Kelly P, Tuberculosis patients’ knowledge and beliefs about tuberculosis: a mixed methods study from the Pacific Island nation of Vanuatu. BMC Public Health. 2014;14:467. 10.1186/1471-2458-14-467 24885057 PMC4033676

[27] Kurbatova EV, Taylor A, Gammino VM, Bayona J, Becerra M, Danilovitz M, Predictors of poor outcomes among patients treated for multidrug-resistant tuberculosis at DOTS-Plus projects. Tuberculosis (Edinb). 2012;92:397–403. 10.1016/j.tube.2012.06.003 22789497 PMC4749016

[28] Shin S, Livchits V, Connery H, Shields A, Yanov S, Yanova G, Effectiveness of alcohol treatment interventions integrated into routine tuberculosis care in Tomsk, Russia. Addiction. 2013;108:1387–96. 10.1111/add.12148 23490304 PMC3710528

[29] Dibben MR, Morris SE, Lean ME. Situational trust and co-operative partnerships between physicians and their patients: a theoretical explanation transferable from business practice. QJM. 2000;93:55–61 and. 10.1093/qjmed/93.1.55 10623783

[30] Lee Y-Y, Lin JL. The effects of trust in physician on self-efficacy, adherence and diabetes outcomes. Soc Sci Med. 2009;68:1060–8. 10.1016/j.socscimed.2008.12.033 19162386

[31] Safran DG, Montgomery JE, Chang H, Murphy J, Rogers WH. Switching doctors: predictors of voluntary disenrollment from a primary physician’s practice. J Fam Pract. 2001;50:130–6 . 11219560

[32] Holtz TH, Lancaster J, Laserson KF, Wells CD, Thorpe L, Weyer K. Risk factors associated with default from multidrug-resistant tuberculosis treatment, South Africa, 1999–2001. Int J Tuberc Lung Dis. 2006;10:649–55 . 16776452

[33] World Health Organization. AUDIT, The Alcohol Use Disorders Identification Test: guidelines for use in primary care. 2nd ed. WHO/MSD/MSB/01.6a. Geneva: The Organization; 2001.

[34] Lalor MK, Greig J, Allamuratova S, Althomsons S, Tigay Z, Khaemraev A, Risk factors associated with default from multi- and extensively drug-resistant tuberculosis treatment, Uzbekistan: a retrospective cohort analysis. PLoS ONE. 2013;8:e78364 and. 10.1371/journal.pone.0078364 24223148 PMC3819387

