# Supplementary material for: Factors Associated with Loss to Follow-up during Treatment for Multidrug-Resistant Tuberculosis, the Philippines, 2012–2014
Source: Emerg Infect Dis. 2016 Mar;22(3):491–502. doi: 10.3201/eid2203.151788 (PMC4766881; doi:10.3201/eid2203.151788)
Supplement: Technical Appendix — Multivariable analysis used to assess factors associated with loss to follow-up during treatment for multidrug-resistant tuberculosis, the Philippines, 2012–2014. [file 15-1788-Techapp-s1.pdf]

Article DOI: http://dx.doi.org/10.3201/eid2203.151788

Factors Associated with Loss to Follow-up during Treatment for Multidrug-Resistant Tuberculosis, the Philippines, 2012–2014

Technical Appendix

Multivariable Analysis

We used multivariable analysis strategy as described in Kleinbaum et al. (*1*). The initial multivariable model included covariates having epidemiologic, biologic, or statistical associations with the dependent variable. The number of variables we were able to include in the initial model was restricted by the sample size, thus we selected 15 variables from >500 variables collected in the database. Investigators evaluated results of the univariate analysis and discussed and agreed on the selection of variables representing all levels of the theoretical framework based on the above mentioned criteria for inclusion in the initial multivariable model. Our initial model included the following variables: age, tobacco smoking, alcohol abuse, summary score on general TB knowledge, summary score on self-efficacy, summary score on social support from the family and friends, receiving any financial assistance from the treatment center, summary score on trust in/rapport with/support from physicians and nursing staff, summary score on information and support from health center staff, use of TB program funds for paying for travel to the treatment center (during intensive phase [IP] of treatment), self-rated severity of vomiting, self-rated severity of dizziness, cost of travel to the Treatment Center [TC] during IP, no time to go for treatment as a major challenge when traveling to the treatment center (IP), patient did not have anyone to go with to TC as a major challenge when traveling to the treatment center (IP).

After selecting variables for the initial model, we evaluated for effect modification in the full initial model. We included the following interaction terms: summary score on general TB knowledge*summary score on self-efficacy; receiving any financial assistance from the treatment center*cost of travel to TC (IP); summary score on trust in/rapport with/support from physicians and nursing staff*summary score on information and support from health center staff. Neither interaction term was statistically significant (based on the Wald test). Statistically non-significant interaction terms mean that association of the summary score on general TB knowledge and loss to follow-up did not vary by the level of the summary score on self-efficacy; association of receiving any financial assistance from the treatment center and loss to follow-up did not vary by the level of the cost of travel to the Treatment Center during intensive phase of treatment; association of the summary score

on trust in/rapport with/support from physicians and nursing staff did not vary by the level of the summary score on information and support from health center staff. All interaction terms were dropped from the model.

Next, we assessed confounding. For this we fitted a “gold standard model” with 15 variables selected as described above. Since this study was hypothesis generating, rather than hypothesis testing, we did not have a “true” main exposure variable, and thus for the purpose of confounding assessment we repeated confounding assessment three times with three variables chosen as main exposure variable for purpose of this analysis (receiving financial assistance from TB program, alcohol use, and self-efficacy score). For each of the “main exposure variables,” we dropped from the full model one variable at a time and evaluated if change in point estimate for the “main exposure variable” is greater than 15% from that in the full model. We did not identify confounding variables in this evaluation.

Finally, we performed manual backward elimination with the purpose of improving precision in the multivariable model and dropped variables that did not have statistically significant association with loss to follow up. We assessed for collinearity among variables in the initial and final models; a variance inflation factor >5 or a maximum condition index >50 were considered evidence of collinearity. No collinearity among variables was detected.

Reference

1. Kleinbaum DG, Klein M, Pryor ER. Logistic regression: a self-learning text: New York: Springer-Verlag; 2002. p. 161–226.
